# Additive effects of gastric volumes and macronutrient composition on the sensation of postprandial fullness in humans

**DOI:** 10.1038/ejcn.2014.194

**Published:** 2014-09-17

**Authors:** L Marciani, E F Cox, S E Pritchard, G Major, C L Hoad, M Mellows, M O Hussein, C Costigan, M Fox, P A Gowland, R C Spiller

**Affiliations:** 1 NIHR Nottingham Digestive Diseases Biomedical Research Unit, Nottingham Digestive Diseases Centre, University of Nottingham, Nottingham, UK; 2 Sir Peter Mansfield Magnetic Resonance Centre, School of Physics and Astronomy, University of Nottingham, Nottingham, UK

## Abstract

**Background/Objectives:**

Intake of food or fluid distends the stomach and triggers mechanoreceptors and vagal afferents. Wall stretch and tension produces a feeling of fullness. Duodenal infusion studies assessing gastric sensitivity by barostat have shown that the products of fat digestion have a greater effect on the sensation of fullness and also dyspeptic symptoms than carbohydrates. We tested here the hypothesis that fat and carbohydrate have different effects on gastric sensation under physiological conditions using non-invasive magnetic resonance imaging (MRI) to measure gastric volumes.

**Subjects/Methods:**

Thirteen healthy subjects received a rice pudding test meal with added fat or added carbohydrate on two separate occasions and underwent serial postprandial MRI scans for 4.5 h. Fullness was assessed on a 100-mm visual analogue scale.

**Results:**

Gastric half emptying time was significantly slower for the high-carbohydrate meal than for the high-fat meal, *P*=0.0327. Fullness significantly correlated with gastric volumes for both meals; however, the change from baseline in fullness scores was higher for the high-fat meal for any given change in stomach volume (*P*=0.0147), despite the lower energy content and faster gastric emptying of the high-fat meal.

**Conclusions:**

Total gastric volume correlates positively and linearly with postprandial fullness and ingestion of a high-fat meal increases this sensation compared with high-carbohydrate meal. These findings can be of clinical interest in patients presenting with postprandial dyspepsia whereby manipulating gastric sensitivity by dietary intervention may help to control digestive sensations.

## Introduction

The main functions of the stomach are to receive and accommodate food, break it down chemically and mechanically and deliver the digesta to the small intestine. This process is regulated by the central and enteric nervous system and neuroendocrine cell networks within a chain of ingestive (cognitive, sensory) and postingestive (nutrient feedback, metabolic responses) satiation and satiety mechanisms.^1, 2, 3, 4, 5, 6^ The sense of fullness and satiation increases with the volume of food ingested^7^ and a linear relationship is present between these sensations and gastric volumes on 3D ultrasound and magnetic resonance imaging (MRI) using model liquid meals.^8, 9, 10^ In addition to meal volume, the macronutrient composition and oro-sensory aspects of the meal have effects on fullness and satiation.^11^ Recent MRI studies have detailed independent effects of volume and caloric load on gastric motor function.^12^ However, it is clear that satiation cannot be explained by changes in gastric relaxation, emptying and nutrient delivery to the small bowel alone.^13^ Gastric sensation also has an important role in determining how much is eaten at a meal. Intake of food or fluid distends the stomach and triggers mechanoreceptors and vagal afferents.^14^ Increased wall stretch and tension produces a feeling of fullness^15,16^ and reduces short-term food intake.^17^ Similar sensations can be induced by gradual distension of an intra-gastric balloon and this has been shown to inhibit neural responses in the amygdala and insula in brain imaging studies; areas that are closely involved in the regulation of appetite and feeding.^18^ In contrast, the stomach does not sense the caloric and nutrient content of food. Rather, nutrient sensing occurs when the products of digestion are absorbed by the small bowel. Simple sugars, amino acids and lipids trigger the release of peptide hormones that directly, and indirectly *via* vagal afferents, regulate gastric function, satiation and satiety.^17,19,20^

Physiological studies that combined duodenal infusion of different nutrients with assessment of gastric sensitivity during stepwise distension of an intra-luminal bag by electronic barostat have shown that the products of fat digestion have a greater effect on the sensation of fullness and also dyspeptic symptoms such as nausea than carbohydrates or proteins.^21, 22, 23^ Excessive fullness, early satiation and nausea after meals are associated with impaired relaxation (‘accommodation') of the stomach in patients with functional dyspepsia^24^ who are also hypersensitive to intra-duodenal fat.^25,26^ These findings suggest that gastric volume and intestinal nutrient feedback have independent effects on the sensations of fullness and satiation. However, the relative contributions of and interactions between these two factors in regulating gastric sensation have not been well described for normal meals because existing investigations are either highly invasive or cannot provide accurate measurements of gastric volume under physiologic conditions.

We hypothesized that gastric volume and meal composition have additive effects on the sensation of postprandial fullness. To investigate this, we performed a randomized, crossover trial to examine the relationship between gastric volumes and the sense of fullness after test meals high in fat or high in carbohydrate in healthy volunteers using MRI: a technology that provides non-invasive measurement of gastric volume change and meal emptying.^27^

## Subjects and methods

### Study design

This study was approved by the University of Nottingham Medical School Research Ethics Committee and all participants gave informed written consent. Eighteen healthy volunteers (nine male and nine female) were enrolled and participated to this study. Previous studies showed significant differences in gastric emptying due to meal characteristics using 8–12 subjects, 18 were recruited to allow for possible dropouts. Five did not finish one of the study meals within the time required and were excluded. Thus, 13 healthy volunteers (8 male and 5 female, 20.8±0.2 years old, with a body mass index of 22.6±0.5 kg/m^2^) completed the study successfully. The participants were apparently healthy and with no contraindication for MRI scanning. They were asked to avoid alcohol and concomitant medications for 24 h, caffeine and strenuous exercise for 18 h and to have a light, non-fatty meal on the evening before the study day. They were asked to fast after this meal until the experimental session the next morning.

The participants attended on two separate occasions ~7 days apart, with each study day lasting ~6.5 h. Subjects were scanned to obtain baseline images of the stomach. Baseline sensation was assessed by visual analogue scale (VAS) scores^28, 29, 30^ (*T*=−45 min). One of two test meals (*T*=−30 min) was then ingested. Subjects were scanned again at *T*=0 min and every 45 min after that with the final scan at *T*=270 min. Immediately after every scan, the volunteers' fullness VAS scores were collected. The two meals were given using a Latin Square design to avoid order effects.

Two test meals were used in this study. They both had, as a common base, the same creamed rice pudding test meal that we have used in previous studies.^31,32^ This consisted of 220 g Sainsbury's creamed rice pudding (Sainsbury, London, UK), 34 g Robertson seedless raspberry jam (Robertsons, Addlestone, UK) eaten with a drink of 100 ml Sainsbury's smooth orange juice from concentrate (Sainsbury). The high-carbohydrate meal contained an additional 50 g of Maxijul powder (Nutricia, Trowbridge, UK), a glucose polymer nutritional supplement. The high-fat meal contained instead an additional 22 g of Sainsbury's double cream (Sainsbury). The macronutrient composition of the two meals is shown in Supplementary Table 1. Accordingly, the high-carbohydrate meal contained 47 g of carbohydrate more than the high-fat meal, which by contrast contained 10 g fat more. The meals were designed to be equally palatable but the overall calorie content was 18% higher in the carbohydrate than the high-fat meal and calorie density was also higher for the carbohydrate meal (5.4 kJ/ml) than for the fat meal (4.3 kJ/ml).

Immediately after each scan, the subjects marked on a VAS their feeling of fullness. The VAS was 100 mm long and each individual score was later measured in mm. The anchors of the VAS were from ‘not full' to ‘extremely full'.^33^ The mean fullness scores were plotted against time for each meal and the area under the curves (AUCs) calculated as above.

### Magnetic resonance imaging

MRI was carried out using a Philips Achieva 1.5T whole-body MRI scanner (Philips, Best, The Netherlands). Volunteers were positioned supine in the scanner with an abdominal 4-element receiver coil placed around the abdomen. At each time point, a transverse balanced turbo field echo was acquired across stomach to measure gastric volumes. This had 24 slices with no gap between them, matrix 160 × 179, in-plane resolution 1.56 × 1.56 mm^2^, slice thickness 10 mm, flip angle 45°, repetition time 2.4 ms, echo time 1.19 ms. The images were acquired under an expiration breath hold of 11 s to minimize respiratory motion artefacts. The subjects spent supine in the magnet only a few minutes at each time point and for the rest of the time they were instructed to sit quietly, upright, in the volunteers' lounge near the scanner room.

### Data analysis and statistical methods

Measurements of the volume of the meal and of the gas in the stomach were carried out manually by tracing regions of interest on each slice using the Analyze6 software (Biomedical Imaging Resource, Mayo Foundation, Rochester, MN, USA) and summing across the slices to determine the total gastric volume. The averaged data sets of volume against time were then analysed by calculating the time for half emptying (T50%) following recently improved gastric emptying modelling^34,35^ by fitting the data to equation (1)


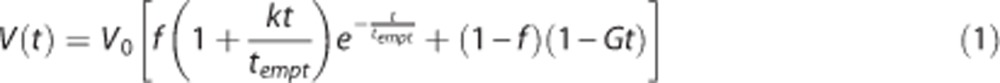


where *V*_0_ is the gastric volume at time 0 and *f*, *k*, *t*_empt_ and *G* are fitting parameters.

The AUC was also calculated with a classic trapezoidal method as overall integration over the study day.

The dependence of fullness on gastric volumes was assessed at individual level using the absolute values and reporting slope, *R*^2^ and *P*-value of the individual linear regressions. However, clinical relevance of sensation is very often not related to absolute values, which can be very different between subjects, but more closely to how much a given change in a physiological parameter relates to a reported change in sensation. To this effect, the changes from baseline (Δ) in fullness scores were plotted against the mean changes from baseline (Δ) in total gastric volumes at corresponding scan time points postprandially and up to 180 min, which was the point after which the stomach volumes returned to baseline.

Statistical analysis was carried out using Prism 5.04 (Graph Pad Software Inc., San Diego, CA, USA). The data were initially tested for normality using the Shapiro Wilk test. Some of the data were not normally distributed even after log transform hence two-way ANOVA was not used. Comparisons between time points (Bonferroni corrected) for the two meals and between the AUCs for the two meals were performed using two-tailed, paired *t*-test (normal data) or Mann–Whitney *U*-test (non-normal data). Correlation was tested using Pearson's test. For all data, *P*<0.05 was considered as statistically significant. The results are given as mean±s.e.m.

## Results

**Figure 1:**
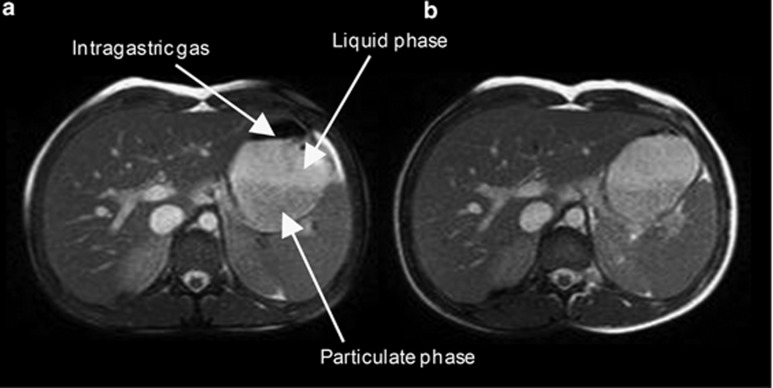
Representative example MRI images of the stomach of a volunteer taken at the first time point after eating (**a**) the high-carbohydrate and (**b**) the high-fat meal on two separate occasions. In the images of the stomach the less intense lower portion belongs to the particulate phase of the meal while the brighter upper phase belongs to a fluid layer. The black part on the top of the stomach is a pocket of intra-gastric gas.

The T2-weighted balanced turbo field echo MRI images in both meals showed early postprandial sedimentation of a dark particulate phase of the meals at the bottom of the stomach with a bright upper layer of fluid (Figure 1). Layering of fat from the aqueous phase was not seen in these T2-weighted images with the low- or high-fat meal.

**Figure 2:**
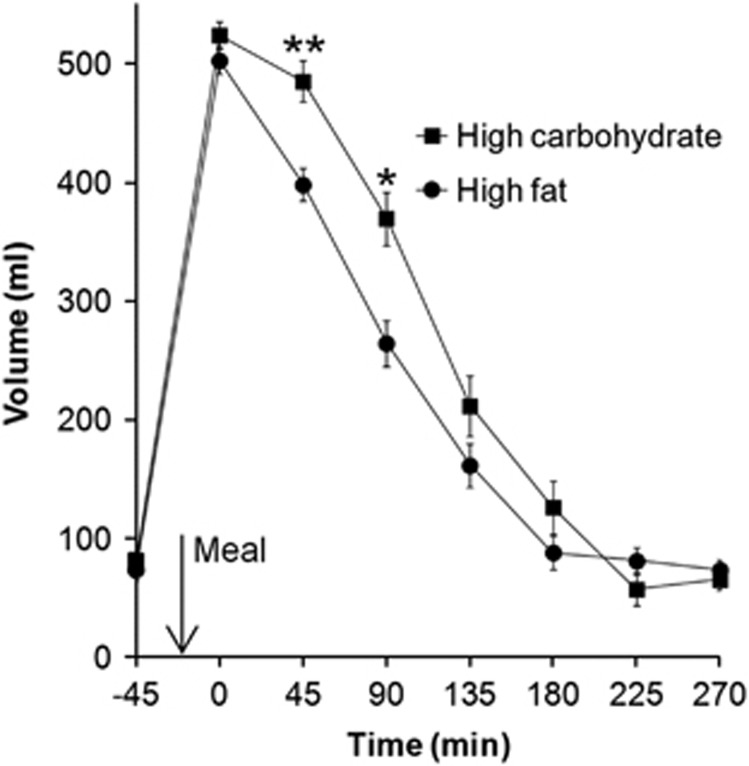
Total gastric volumes (chyme plus gas) with time after eating the high-carbohydrate and the high-fat meal. Values are mean±s.e.m., *n*=13. *P<0.05, **P<0.001 between time points, paired *t* test, Bonferroni corrected.

The dynamic change in gastric volumes over time is shown in Figure 2. As expected, the fasted stomach contained a small volume of gastric secretions with no difference between the two study days (*P*=0.67). Gastric volumes increased after the meal to just over 500 ml with no significant difference between meals (*P*=0.12) and then declined back to baseline within 3 h. Gastric emptying was significantly slower for the high-carbohydrate meal than for the high-fat meal as indicated by the time to half empty T50% (122±10 min versus 99±8 min, respectively, *P*=0.0327) and by the AUCs (83 195±3718 versus 70 711±3182 ml min, respectively, *P*=0.0016). Gastric volumes at time points 90 and 135 min were significantly different (Bonferroni corrected).

**Figure 3:**
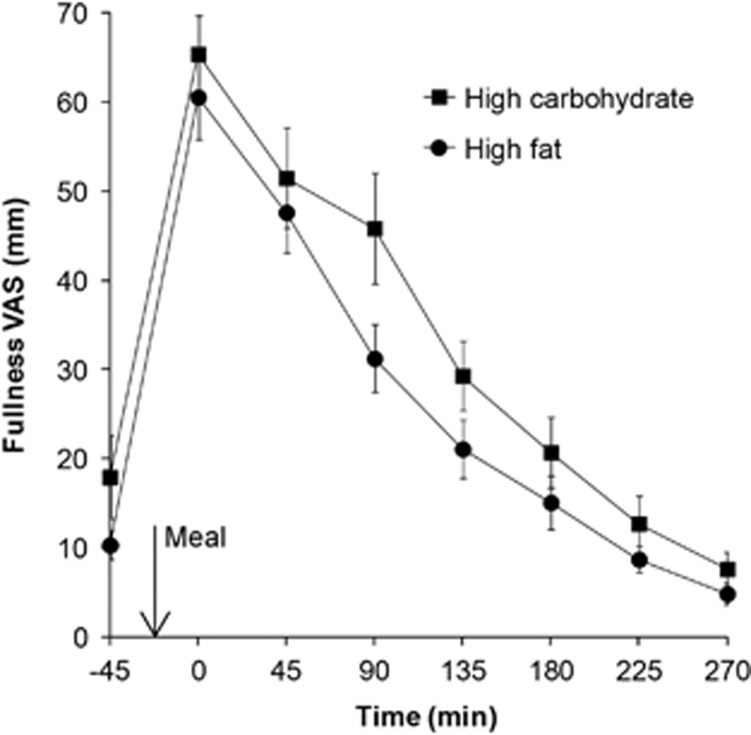
VAS feelings of fullness measured after eating the high-carbohydrate and the high-fat meal. Values are mean±s.e.m., *n*=13.

The change in subjects' reported fullness VAS scores over time is shown in Figure 3. There was no difference at baseline (*P*=0.51). Fullness increased threefold to fourfold after both meals and returned to baseline by the end of the study day. Fullness was higher after the high-carbohydrate meal than the high-fat meal (AUC 107 17±1009 versus 8631±834 mm min, respectively, *P*=0.021). Fullness at the time points at time 90 and 135 min was significantly different with *P*<0.05 uncorrected for multiple comparison; however, the Bonferroni correction removed the significance from the difference due to variability.

The absolute values of fullness were significantly correlated with gastric volumes for both meals (Supplementary Figure 1). Fullness and gastric volumes were correlated at individual level (Supplementary Table 2). The fullness/volume individual slopes for the carbohydrate meal (0.098±0.015 scores/ml) and for the fat meal (0.12±0.01 scores/ml) were not significantly different between meals (*P*=0.1600). This was despite the differences between meals.

**Figure 4:**
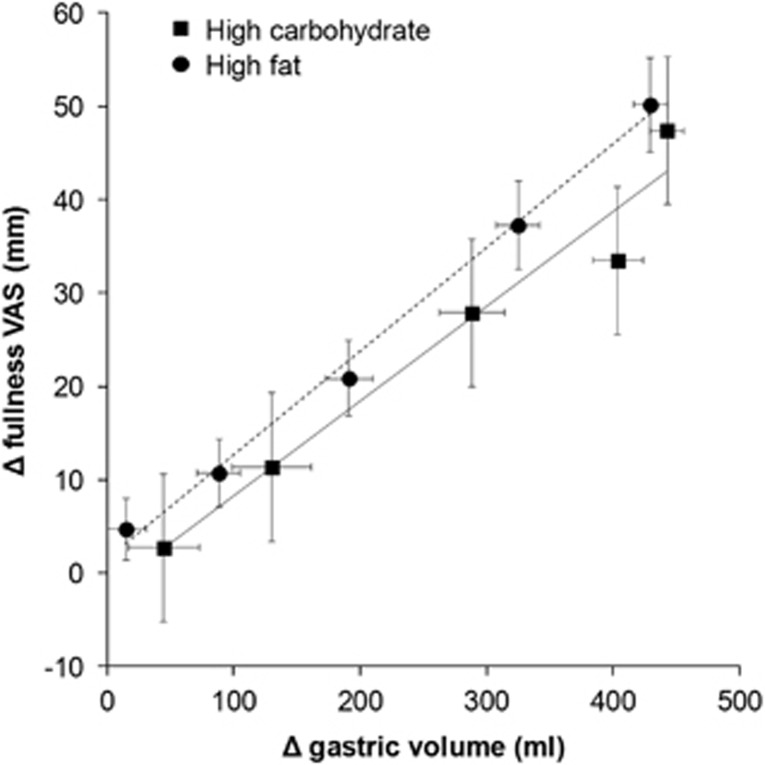
Changes from baseline (Δ) in fullness VAS scores plotted against the mean changes in total gastric volumes at corresponding scan time points postprandially and up to 180 min (the point after which the stomach volumes returned to baseline). The solid and dotted lines are respectively the linear regression lines of best fit for the high-carbohydrate meal (*R*^2^=0.97, *P*=0.0026) and the high-fat meal (*R*^2^=0.99, *P*=0.0002). Values are mean±s.e.m., *n*=13.

To assess the relationship between gastric volumes and fullness, the mean changes from baseline (Δ) were calculated for each individual at each scan time point after the meal until the stomach volumes returned to baseline (*T*=180 min). For each individual, a Pearson's correlation *R*^2^ between Δ fullness and Δ gastric volume was calculated. These data show that fullness and gastric volumes were highly correlated with a mean *R*^2^=0.83±0.04 for the high-carbohydrate meal and *R*^2^=0.94±0.01 for the high-fat meal. The individual data were then averaged by time point and by meal and these data are shown in Figure 4. The lines of best fit show a linear relationship between fullness and gastric volume for both the high-carbohydrate meal (*R*^2^=0.97, *P*=0.0026) and the high-fat meal (*R*^2^=0.99, *P*=0.0002). There was no significant difference between the gradient of the linear relationship between meals (*P*=0.5214). However, the line of fit for the high-fat meal is shifted up compared with the high-carbohydrate meal with a significant difference between intercepts (*P*=0.0147), indicating higher fullness for the high-fat meal at any given stomach volume. This occurred despite the lower energy content and faster gastric emptying of the high-fat meal.

## Discussion

This non-invasive MRI study demonstrates the important interaction between the volume and macronutrient composition of a meal on gastric motor and sensory function in healthy subjects under physiological conditions.

Gastric volume measured immediately after ingestion of the two test meals was almost identical; however, half gastric emptying time of the meal was 19% faster following the high-fat meal compared with the high-carbohydrate meal. This could well be related to the proportionately lower energy content (18%) and lower energy density of the high-fat compared with the high-carbohydrate meal.^13,36^ Effects of osmolality on gastric emptying may also be important because a previous MRI study reported similar differences in the gastric emptying curve even when the calorie load was strictly controlled in 500 ml fat and glucose solutions.^10^ During gastric emptying MRI showed that both test meals layered in the stomach, with a liquid phase collecting above the solid phase. Subsequently, as we have described for other test meals,^37^ the liquid component was seen to empty first (gastric sieving) and arrived at the duodenum before the solids. The composition of this liquid layer is not certain; however, unlike our studies of oil in liquid emulsions where separate fat and aqueous layers were observed in the stomach,^38^ no fat layering was observed on T2-weighted scans. This is likely to relate to a combination of buffering of gastric acid by the protein in the meal and the stabilizers in the double cream preventing ‘cracking' into separate lipid and water phases.^33^

Consistent with previous MRI studies,^8, 9, 10^ the sensation of fullness decreased after the meals as food emptied from the stomach. This was closely correlated with the decrease in gastric volume. The individual and average slopes of regression of the fullness versus total gastric volume were not significantly different despite the differences between meals. However, when considering how much a change in gastric volume impacted on the sensation of fullness, the line of fit for fullness versus gastric volumes was shifted up in the high-fat meal compared with the high-carbohydrate meal. This indicates that subjects experienced a greater degree of fullness at any given gastric volumes for the high-fat compared with the high-carbohydrate meal. Such results cannot be explained by differences in gastric emptying, which was quicker, or energy load, which was lower for the high-fat meal. Rather, this is evidence that the high-fat meal increased visceral sensitivity to gastric distension compared with the high-carbohydrate meal. Further, these findings support the study hypothesis that volume and meal composition have additive effects on the perception of postprandial fullness. The mechanism by which fat increases visceral sensitivity has been studied. The peptide hormone cholecystokinin released by the duodenum in response to products of lipid digestion has been shown to enhance fullness, satiation and other sensations induced by distension of an intra-gastric balloon^39^; an effect that is partially blocked by administration of loxiglumide a specific cholecystokinin antagonist.^26^ Other peptides such as glucagon-like peptide 1 and polypeptide YY are also involved in nutrient feedback and satiety signalling.^40, 41, 42^

The key strength and novel contribution of this study was the use of validated, non-invasive MRI technology to acquire serial, high-resolution measurements of gastric volumes in subjects under physiologic conditions.^27^ MRI measures of total gastric volumes provide a direct measurement of gastric relaxation and contraction. This is a good approximation of meal accommodation although a true measure of gastric tone requires concomitant intra-gastric volume and pressure measurements.^12^ Two different meals were used, one high in fat and one high in carbohydrate, which are known to have differing hormonal, metabolic and satiation responses,^43,44^ properties that allowed us to test the study hypothesis. One limitation is that the two test meals were not equicaloric nor matched for osmolality or taste; however, they were equally palatable. Perfect calorie matching would probably have increased the effect size. However, our ability to measure accurately the gastric volumes provided in the final analysis convincing data that fat increases visceral sensitivity. The lack of a control meal made it difficult to deconstruct more precisely the additive role of macronutrients and volume. Another limitation was the relatively high percentage of subjects (30%) that did not finish the meal in the time allowed leading to exclusion from the Per Protocol analysis. Rather than palatability, the relatively large size and rich calorie content of the meal could have played a role because failure to complete occurred with both the meals; we could have avoided this by allowing more time in the protocol to finish eating the meal. No meal preference assessment was made. We did not have the resources to carry out serial blood sampling and hormone assays; however, future studies should include sampling of peptide hormone levels to allow assessment of the role of cholecystokinin and other gastrointestinal peptides as well as assessment of *ad libitum* test meal consumption to assess whether the different effect on satiation leads to changes in subsequent food consumption.

In conclusion, we have shown that total gastric volume correlates positively and linearly with postprandial fullness and that ingestion of a high-fat meal increases this sensation compared with high-carbohydrate meal. Unfortunately, these findings may not help to reduce short-term food intake and facilitate weight control as high-fat foods usually have higher energy density than high-carbohydrate foods and so the volume needed to take on the same calorie load would also be lower. Moreover, high-fat foods are often highly palatable. These issues highlight the complex interplay of hedonic and physiological signalling that drives food (over)consumption.^4^ Our findings could also be of clinical interest in patients presenting with postprandial dyspepsia. Here, manipulating gastric sensitivity by dietary intervention may help to control digestive sensations. One clinical and physiological trial has shown a reduction in reflux sensations of 40% in patients on an high-carbohydrate compared with equicaloric, high-fat diet,^45^ and similar effects could be inferred from our findings in healthy volunteers and in functional dyspepsia.^25,26^
