# Supplementary material for: Additive effects of gastric volumes and macronutrient composition on the sensation of postprandial fullness in humans
Source: Eur J Clin Nutr. 2014 Sep 17;69(3):380–4. doi: 10.1038/ejcn.2014.194 (PMC4351404; doi:10.1038/ejcn.2014.194)
Supplement: Supplementary Information [file ejcn2014194x1.pdf]

Supplementary Table 1. Macronutrient composition of the two study meals

	Amount (g)	Energy (kJ)	Protein (g)	Total carbohydrate (g)	Carbohydrate of which sugars (g)	Fat (g)	Fiber (g)	Salt (g)
High carbohydrate meal								
Rice pudding	220	887	6.8	37.2	22.4	3.7	0.4	0.1
Raspberry jam	34	353	0.2	20.5	18.7	0.03	1.0	0.03
Orange juice	100	179	0.5	9.1	9.1	0.1	0.1	trace
Glucose polymer	50	808	0	47.5	4.3	0	0	trace
Total for high carbohydrate meal	404	2226	7.5	114.3	54.6	3.9	1.6	0.1
High fat meal								
Rice pudding	220	887	6.8	37.2	22.4	3.7	0.4	0.1
Raspberry jam	34	353	0.2	20.5	18.7	0.03	1.0	0.03
Orange juice	100	179	0.5	9.1	9.1	0.1	0.1	trace
Double cream	22	403	0.4	0.6	0.6	10.5	0	trace
Total for high fat meal	376	1822	7.9	67.4	50.8	14.3	1.6	0.1

Supplementary Table 2. Individual linear regression of absolute values of fullness and gastric volumes for each subject and meal

	Slope	R^2	p	Slope	R^2	p
Subject	***Carbohydrate meal***			***Fat meal***		
1	0.072	0.6944	0.0102	0.173	0.9457	0.0001
2	0.063	0.6358	0.0178	0.096	0.9334	0.0001
3	0.104	0.7886	0.0032	0.059	0.6504	0.0156
4	0.085	0.8568	0.0010	0.073	0.8778	0.0006
5	0.089	0.5336	0.0396	0.139	0.9078	0.0003
6	0.079	0.6218	0.0200	0.048	0.7759	0.0039
7	0.137	0.7056	0.0090	0.179	0.9552	0.0001
8	0.062	0.7466	0.0057	0.130	0.9412	0.0001
9	0.170	0.9332	0.0001	0.126	0.7578	0.0049
10	0.004	0.0018	0.9209	0.090	0.9367	0.0001
11	0.198	0.9048	0.0003	0.184	0.7183	0.0079
12	0.066	0.8124	0.0022	0.110	0.9797	0.0001
13	0.147	0.8172	0.0021	0.137	0.9259	0.0001

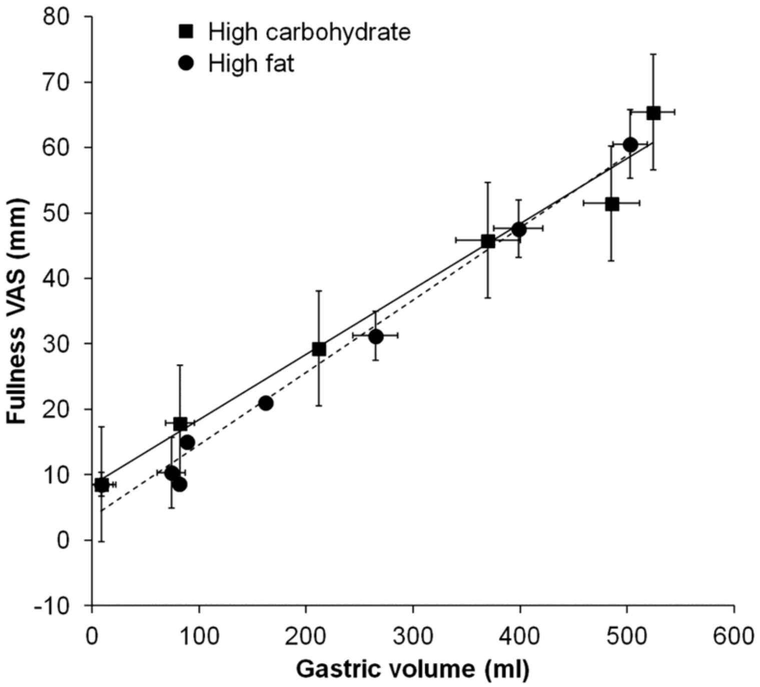

Supplementary Figure 1:

Absolute values of fullness visual analogue scale (VAS) scores plotted against the absolute values of total gastric volumes at corresponding scan time points. The solid and dotted lines are respectively the linear regression lines of best fit for the high carbohydrate meal (R^2=0.98, P<0.0001) and the high fat meal (R^2=0.98, P<0.0001). Values are mean ± s.e.m., n=13.
